# Supplementary material for: Added body mass alters plantar shear stresses, postural control, and gait kinetics: Implications for obesity
Source: PLoS One. 2021 Feb 5;16(2):e0246605. doi: 10.1371/journal.pone.0246605 (PMC7864670; doi:10.1371/journal.pone.0246605)
Supplement: Attachment — Submitted filename: Weight_Distribution.docx [file pone.0246605.s001.docx]

The authors present an interesting study that investigated the effect of increased body mass and altered mass distribution associated with obesity on plantar shear that may putatively lead to physical functional deficits. While the results are potentially informative and may provide a meaningful contribution to this rapidly developing field, there were a few aspects of the paper that were unclear as currently written.

1. Authors justified of excluding female based on distinct body fat accumulation tendency between male and female. However, in the current study objective of the authors is to compare effect of weight distribution conditions (i.e., Forward distributed vs Evenly distributed vs Normal weight) and not within subject differences. Therefore, in my opinion given justification to exclude female is weak and raises issues related to generalizability of the results.
2. Authors need to double check for the typo. For instance, in the abstract, there were typo such as (..).
3. Once the abbreviation of a term is provided than authors do not need to provide it again. For instance in the abstract authors provided abbreviation for evenly distributed (EV), or frontloaded vest (FV) twice.
4. In the methodology, if the experiments were conducted according to declaration of Helsinki then authors should mention it in the methodology.
5. In the figure, significant differences are not represented. The figures should be self-explanatory, and readers should not be needed to refer text or able to identify group significant differences. Providing significant differences through arrow would make it convenient for the readers to understand. Additionally, in figure 1, authors should have represented the AP_Static or ML_Static as AP Static or ML Static.
6. In discussion, references are required for the statement, “A few previous studies have indirectly investigated the effects of mass on medial longitudinal arch deformation.”
7. The following sentence in the discussion was confusing and need to be rephrashed, “This reduced plantar cutaneous sensitivity has been associated with increased postural sway [7], thus our decision to simultaneously investigate postural sway measures.”
8. It was not clear if the authors want to indicate association or causation in the following statement in discussion, “Previous load carriage studies show that healthy young individuals walking with a heavy backpack decrease stride length and increase double support time through decreased hip and knee sagittal plane angular displacement [44].”
